# Simple water‐based tacrolimus enemas for refractory proctitis

**DOI:** 10.1002/jgh3.12280

**Published:** 2019-11-14

**Authors:** Sasha R. Fehily, Felicity C. Martin, Michael A. Kamm

**Affiliations:** ^1^ Department of Gastroenterology St Vincent's Hospital Melbourne Victoria Australia; ^2^ University of Melbourne Melbourne Victoria Australia

**Keywords:** basic science, endoscopy, experimental models and pathophysiology, pathology

## Abstract

**Background and Aims:**

Rectal ulcerative colitis (UC) and Crohn's disease (CD) often do not respond to conventional therapies. Oral and suppository tacrolimus are effective but often poorly tolerated or are complex to formulate. Tacrolimus is topically active, water soluble, and has minimal systemic toxicity when administered rectally; we therefore tested a simple tap water‐based enema formulation.

**Methods:**

Tacrolimus powder from 1 mg capsules and tap water in a 60 mL syringe were delivered rectally. The primary end‐point was endoscopic response (UC: MAYO score reduction by one point; CD: improvement in ulcer number and severity). Secondary end‐points included endoscopic remission, clinical response, stool frequency, and rectal bleeding.

**Results:**

Seventeen patients [12 UC, five CD, nine female, median age 31 years] with refractory rectal disease were treated. The majority of patients had failed immunosuppressive therapy [88% thiopurine; 71% biologic therapy]. Initial enemas included 1–4 mg tacrolimus daily and 1–3 mg tacrolimus maintenance three times a week for a median of 20 weeks (range 3–204). Concomitant thiopurine or biologic therapy continued. 94% tolerated therapy. Of 12 UC patients, eight (67%) achieved endoscopic remission, one further patient achieved endoscopic response, and median partial MAYO scores decreased (pre:4 vs. post:2; *P* = 0.010). Of five CD patients, three (60%) achieved endoscopic response, two (40%) endoscopic remission, and three (60%) clinical response. Stool frequency, rectal bleeding, and C‐reactive protein levels improved. Strictures became endoscopically passable in all four affected patients. No major adverse events were reported, and four patients had disease flare.

**Conclusions:**

Tacrolimus enemas are easy to prepare, well tolerated, effective, and safe. They should be included in the treatment armamentarium for inflammatory bowel disease‐related refractory proctitis.

## Graphical abstract

Rectal ulcerative colitis and Crohn's disease often do not respond to conventional therapies. Tacrolimus enemas are easy to prepare, well tolerated, effective, and safe. They should be included in the treatment armamentarium for inflammatory bowel disease‐related refractory proctitis.

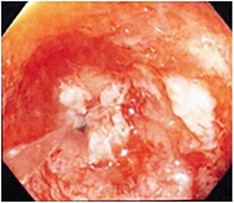

## Introduction

Isolated proctitis occurs in both ulcerative colitis (UC) and Crohn's disease (CD). Typical features include increased stool frequency, bloody diarrhea, urgency, and tenesmus. First‐line therapies include corticosteroids, 5‐aminosalicylic acids (5‐ASAs), or a combination of the two.^1^, ^2^ The disease extent determines the most appropriate topical formulation.^3^ A combination of oral and topical medication is more efficacious than either alone.^4^, ^5^ Treatment escalation for persistent active disease involves immunosuppressive therapy^6^ or biologic therapy, including monoclonal antibodies to tumor necrosis factor alpha (anti‐TNF), integrins, or interleukins (IL)‐12/23. Recent treatment targets focus on “deep remission “.^7^ For both short‐ and long‐term outcomes, data support the early escalation of therapy for unresponsive patients.^8^, ^9^ Despite these advances, many patients do not respond to immunosuppressive or biologic therapies, and effective therapy remains a challenge.

Calcineurin inhibitors (CNIs), including cyclosporine and tacrolimus, have a long history of use in inflammatory bowel diseases (IBD). Cyclosporine is effective in acute severe UC refractory to corticosteroids.^10^, ^11^, ^12^ Oral tacrolimus is an alternative to conventional acute and maintenance therapy in patients with resistant IBD.^13^, ^14^, ^15^, ^16^, ^17^, ^18^ Their oral use is associated with gastrointestinal side effects, hypertension, pruritis, tremor, hyperglycemia, and paraesthesia.^16^, ^19^ More serious adverse reactions include renal impairment, opportunistic infections, nonmelanoma skin cancers, and myelosuppression.^19^, ^20^ Many of these adverse reactions are related to the serum level.^21^ Although cyclosporine and tacrolimus have the same mechanism of action, their molecular characteristics and pharmacokinetic profiles differ, resulting in tacrolimus having a more favorable side effect profile.

CNIs are generally regarded as having poor water solubility, with variable intestinal absorption and unpredictable blood levels. Topical tacrolimus is transdermally absorbed and is therefore used in dermatological conditions, including atopic dermatitis. Low systemic toxicity has been reported with topical formulations.^22^ Suppository and rectal ointment tacrolimus formulations have been proven to have therapeutic efficacy in IBD^23^ but are often poorly tolerated or are complex to formulate. One study included a small number of patients with tacrolimus enemas.^24^

Tacrolimus is highly topically active in a short time, has minimal systemic toxicity when administered rectally, and is soluble in water. We have therefore tested a very simple tap water‐based enema formulation.

## Methods

A review was performed of all patients with refractory IBD‐related proctitis who had been treated with tacrolimus enemas from June 2013 until October 2018. All patients had clinical refractory proctitis defined by at least three relapses a year, or chronic active disease, despite conventional treatment including immunomodulator or biologic therapies. Moderate to severe active proctitis had to be confirmed endoscopically for inclusion.

Patients self‐prepared the tacrolimus enemas in their home environment. To ensure tolerability, daily tacrolimus enemas were started at a dose of 1 mg in 60 mL of water and were then increased to 3 mg in 60 mL water over 7 days. Tacrolimus powder was emptied from 1 mg capsules into a 60 mL Toomey bladder syringe, 60 mL of warm tap water was added, and a soft catheter was used to self‐deliver the solution rectally. Once clinical and endoscopic remission had been achieved, 1–3 mg of maintenance tacrolimus enemas were used thrice a week. Duration of therapy was determined by the treating physician and was individualized according to the patient's condition. Partial compliance was assessed by patient request for further prescriptions.

All patients underwent regular clinical assessment monthly and biochemical (C‐reactive protein [CRP]) monitoring monthly initially and then every 2 months thereafter. The timing of endoscopic monitoring was dictated by achievement of clinical response. The primary end‐point was endoscopic response (UC: MAYO score reduction by one point; CD: decrease in the number and severity of ulcers and the proportion of mucosa inflamed). Secondary end‐points were endoscopic remission (UC: MAYO 0 or 1; CD: absence of ulcers/inflammation), retrospectively derived clinical response (UC: partial MAYO score [bowel frequency, rectal bleeding, and physician global assessment, each rated 0–3]; CD: decrease by 50% in bowel frequency or becoming normal), stool frequency, and percentage rectal bleeding at week 8. Composite endoscopic scores were not used in patients with CD as only limited endoscopic evaluations were undertaken to assess the improvement of proctitis.

Clinical and endoscopic evaluations were undertaken by a single treating physician (MAK). Data of all treated patients were reviewed by two physicians (SRF, FCM), independent from the treating physician, with respect to clinical, biochemical, and endoscopic responses and adverse drug reactions (ADRs).

## Results

**Figure 1:**
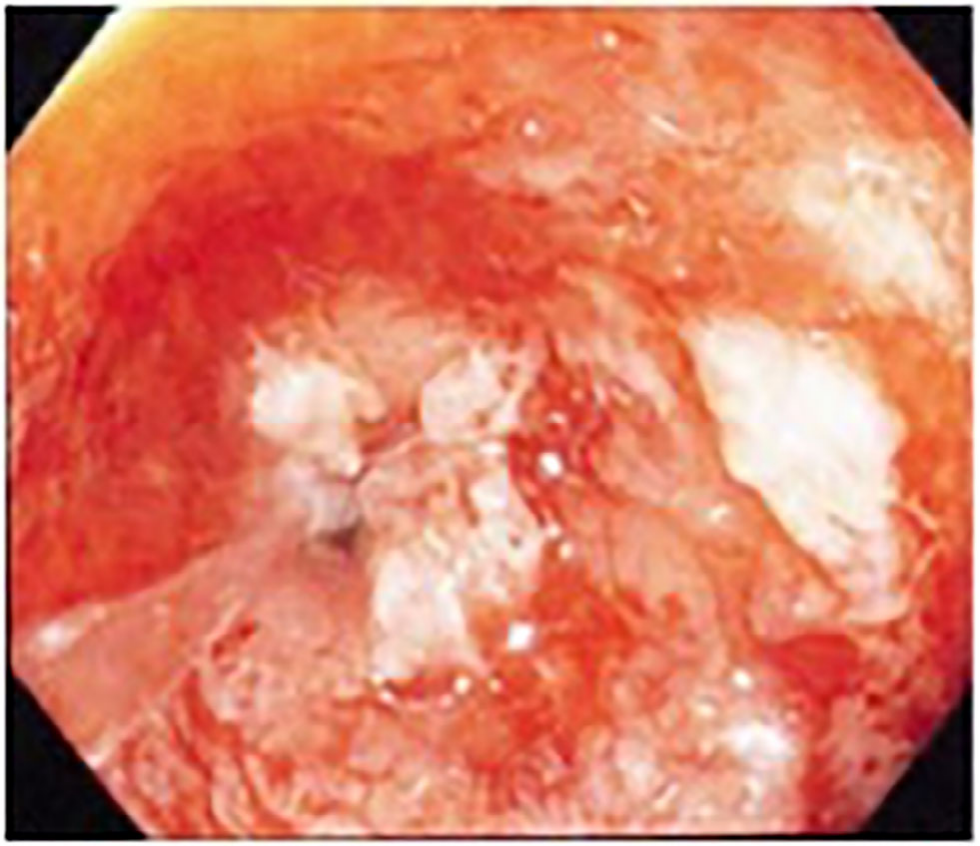
Distal end of a rectal stricture, with pinhole lumen, in a patient prior to tacrolimus enema therapy.

Patient characteristics of all patients are shown in Table 1. Of 17 treated patients, 12 (71%) had UC and 5 (29%) had CD. Median age at diagnosis was 31 years (range: 22–50). Most patients had disease affecting only the left colon or rectum. Four patients had endoscopically impassable inflammatory rectal strictures (Fig. 1).

**Table 1:** Patient details

Gender	Male 8 (47%) Female 9 (53%)
Median age at diagnosis	31 (range 22–50)
IBD type	Ulcerative colitis 12 (71%) Crohn's disease 5 (29%)
Baseline median MAYO score	2 (IQR 2–3)
Baseline median partial MAYO score	4 (IQR 3–5)
Past therapy	
Steroid use	17 (100%)
5‐ASA	14 (82%)
Thiopurine	15 (88%)
Biologic agent	12 (71%)
Other	8 (47%)
Concomitant therapy	
Steroid use	2 (12%)
5‐ASA	6 (35%)
Thiopurine	14 (82%)
Biologic agent	7 (41%)
Other	4 (24%)

All patients were resistant to standard therapies. All patients had been resistant to oral or rectal corticosteroids. Fourteen (82%) patients had been treated with 5‐ASA therapy, 15 (88%) a thiopurine, 12 (71%) a biologic agent, and 8 (47%) other therapies (4 oral or 2 suppository CNI, 1 oral thalidomide and fecal microbiota transplantation). Past biologic agent use included infliximab (44%), adalimumab (39%), and vedolizumab (17%).

Sixteen (94%) patients remained on concomitant IBD therapy: 5‐ASA 6 (35%), thiopurine 14 (82%), biologic agent 7 (41%), other 4 (24%). Of the patients on concomitant biologic therapy, two (29%) patients were on infliximab, two (29%) on adalimumab, and three (42%) on vedolizumab. Two patients on prednisolone were able to rapidly be weaned on commencement of tacrolimus enemas. Eleven patients (65%) continued maintenance dosing after achieving clinical and endoscopic remission at varying doses: 7 (64%) at 3 mg, 3 (27%) at 2 mg, and 1 (10%) at 1 mg.

Sixteen (94%) patients tolerated the enema self‐administration.

In patients with UC, 8 of 12 (67%) achieved endoscopic remission, and one further patient achieved endoscopic response. Response or remission was therefore achieved in 9 of 12 (75%) UC patients. The pretreatment MAYO scores decreased from a median of 2 (IQR 2–3) to 1 (IQR 0–2) posttreatment (*P* = 0.010). The partial MAYO scores significantly improved from 4 (IQR 3–5) to 2 (0–3) posttreatment (*P* = 0.010).

Three of five (60%) patients with CD achieved endoscopic response (decrease in the number and severity of ulcers and the proportion of mucosa inflamed). In terms of secondary outcomes, 40% of patients achieved endoscopic remission (absence of ulcers/inflammation), and 60% achieved clinical response (decrease or normalization of bowel frequency).

**Figure 2:**
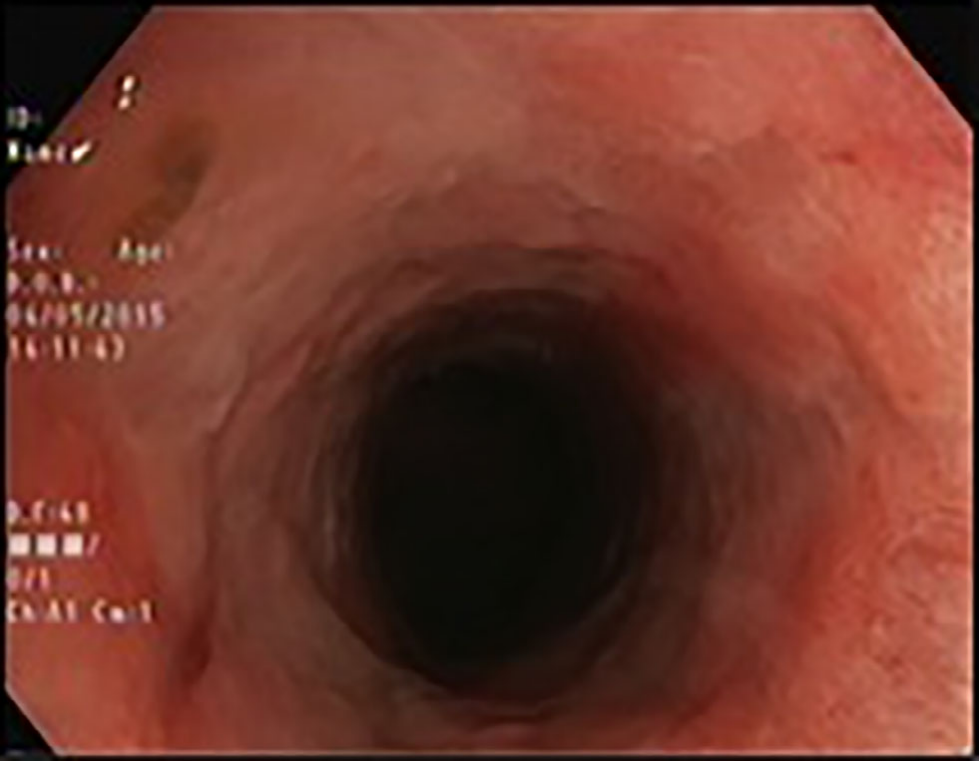
Mild stricture in the same patient after tacrolimus enema therapy.

The entire cohort of patients had significant reductions in median stool frequency (*P* = 0.017) and percentage of rectal bleeding (*P* = 0.009). Nine patients with an elevated CRP experienced a decrease in CRP, and seven patients maintained a normal CRP. Median CRP levels pre‐ and post‐treatment were 11 (0–23) *versus* 0 (0–13), respectively (*P* = 0.019). In all four patients with strictures, the inflammation resolved, and the stricture became endoscopically passable without dilatation (Fig. 2).

Tacrolimus enemas were administered for a median of 20 weeks (range 3–204).

Four patients had a flare of their more proximal disease while receiving topical tacrolimus therapy. All these patients had UC—two with pancolitis and two with left‐sided colitis. All these patients were receiving concomitant immunosuppression with either a thiopurine, a biologic agent, or both.

Three patients experienced pruritus or nausea. The patient experiencing pruritus was able to continue therapy despite the presence of an ADR. For the patients reporting nausea, this ADR was experienced at a tacrolimus dose of 3 mg; for one of these patients, the 2 mg dosage was tolerable, and the other patient required other treatment due to a disease flare.

## Discussion

We have demonstrated that simple water‐based tacrolimus enemas are well tolerated and can be effective in combination with patient's existing therapy for resistant proctitis, particularly in UC. The patient can formulate the enema each evening. The induction dose can be titrated according to tolerance and the therapeutic response. A thrice‐weekly maintenance regimen, based on previous rectal mesalazine maintenance therapy,^25^ effectively maintains remission, with dose titration as needed.

Rectal biopsies 3–5 h of enema administration confirm mucosal absorption. Systemic drug levels are low when tacrolimus is used rectally.^23^, ^24^, ^26^ Blood levels do not rise substantially from a trough level after repeat rectal dosing,^24^ which is why measurement of serum drug levels was not performed.^23^, ^24^, ^26^ However, we cannot exclude that systemic levels in the low therapeutic range were responsible for a few of the systemic effects,^27^ particularly as some patients do have mild systemic symptoms, such as tremor. In these patients, the enema dose can be reduced.

A disease flare, in affected bowel beyond the reach of enemas, occurred in four patients with UC during tacrolimus enema therapy. These patients had previously required multiple immunosuppressive and biologic agents. Pretreatment endoscopy for two of these patients demonstrated pancolitis with more marked inflammation in the rectum; the extent of colonic involvement in these patients is likely to have increased the possibility of therapeutic failure. Two patients with left‐sided disease flare required escalation of therapy, a biologic agent in one patient and subtotal colectomy in the other.

Our small sample size relates to this being a preliminary report, and therefore, further controlled trials are warranted. The limitations of this study include the heterogeneity of the cohort and the variable duration of therapy received. Serum levels of tacrolimus would have been helpful in determining predictors of clinical response and the presence of ADRs.

There are two previous reports on the efficacy of topical tacrolimus in different formulations in patients with refractory proctitis related to IBD. Following a successful prospective pilot study evaluating tacrolimus rectal ointment in ulcerative proctitis by Lawrance et al.,^26^ a randomized multicenter, double‐blind, placebo‐controlled trial was performed by the same group.^23^ Eleven patients received rectal tacrolimus ointment at a dose of 3 mL (0.5 mg/mL), and 10 received placebo ointment, twice daily, for eight weeks. Eight (73%) patients receiving tacrolimus *versus* one (10%) patient in the placebo group achieved a clinical response (*P* = 0.004). Clinical remission was achieved in 45% of the tacrolimus group compared to none in the placebo group (*P* = 0.015). Tacrolimus trough levels were either undetectable, subtherapeutic, or in the low therapeutic range. Adverse effects reported include an upper respiratory tract infection, mild tremor, and headache. There were no associations identified between serum trough levels and the clinical outcome or side effects.

A prospective study of 19 patients with proctitis or left‐sided colitis were treated with 2–4 mg of tacrolimus daily, in the form of suppositories or enemas, for 4 weeks.^24^ Of the 19 patients, 12 used suppositories for proctitis; the remaining 7 patients with left‐sided colitis used enemas to distribute the drug over the larger mucosal area involved. The enema preparation was similar that of our study, with tacrolimus powder emptied from capsules into 100 mL of sterile water. The majority of patients received concomitant oral immunosuppressive therapy. Of the 12 patients with distal UC, 10 experienced clinical improvement after 4 weeks of suppository therapy, and 8 patients had endoscopic improvement. Of the five patients with left‐sided colonic UC, three improved clinically with enemas. The two patients with left‐sided colonic CD, who used tacrolimus enemas, did not experience clinical improvement. No adverse effects were reported, and all patients had a low trough level (<5 μg/L).

This case series demonstrates the efficacy of tacrolimus in a novel formulation that is easy to prepare, effective, tolerable, and safe. Tacrolimus enemas should be included as adjuvant therapy in the treatment armamentarium for refractory proctitis.
